# Beware the painful nerve palsy; neurostenalgia, a diagnosis not to be missed

**DOI:** 10.1007/s11751-012-0143-6

**Published:** 2012-10-04

**Authors:** Jane Halliday, Tim Hems, Hamish Simpson

**Affiliations:** 1 Department of Trauma and Orthopaedics, Edinburgh Royal Infirmary, 51 Little France Crescent, Old Dalkeith Road, Edinburgh, EH16 4SA UK; 2 Department of Trauma and Orthopaedics, Glasgow Royal Infirmary, 84 Glasgow St, Glasgow, G4 0SF UK

**Keywords:** Neurostenalgia, Limb lengthening, Decompression, Radial nerve

## Abstract

We present a case of painful radial nerve palsy following application of a humeral lengthening frame. At re-operation, the radial nerve was found to be compressed against a distal pin. This was re-sited providing immediate pain relief and a gradual resolution of the radial nerve palsy. Pain in association with a nerve palsy should alert the clinician to the possibility of nerve compression that may benefit from urgent decompression.

## Introduction

Neuropathic pain, arising from a lesion of the nervous system, is defined as persistent intractable pain disproportional between the extent of the lesion and the severity of the pain. It is associated with sensory and/or motor disturbance [1]. Neurostenalgia is a neuropathic pain that results from continuing irritation of an anatomically intact nerve by a noxious agent [1]. It may be chronic or acute and is not associated with excess sudomotor or vasomotor activity. Common causes include compression, distortion, ischaemia or tethering of the nerve. Removal of the causative agent typically results in immediate relief of pain and a more gradual recovery of nerve function. If the cause persists, irreversible changes in the affected nerve may occur with the nerve lesion worsening from that of a conduction block to a degenerative lesion [1]. It is therefore important that pain in relation to nerve palsy is recognised and acted upon quickly to prevent significant nerve damage from occurring.

## Case report

A 10-year-old girl underwent application of a humeral lengthening frame of her right humerus to correct severe humeral shortening gradually. The shortening was secondary to osteomyelitis of the right proximal humerus aged 10 weeks. This had left her with a 10-cm discrepancy between the lengths of the right and left humeri, resulting in significant functional difficulties. The lengthening frame was applied with the elbow extended without any intra-operative complications. In recovery, her right hand was warm and well perfused with full sensation and movement of the wrist and small hand joints. On the first post-operative day, however, she complained of severe burning pain in her right arm and hand requiring significant opiates for relief and a pain team review. Examination revealed evidence of a partial right radial nerve palsy; weakness of right wrist extension, an inability to extend the fingers and diminished sensation on the dorsum of the first webspace. She displayed evidence of hyperalgesia with an exaggerated response to pin-prick on the dorsum of the first webspace. There was no mechanical allodynia. All other hand and wrist movements and sensation were intact.

An ultrasound scan of the right arm was performed. This revealed no haematoma or collection. The radial nerve was not visualised.

On the second post-operative day, her symptoms decreased slightly. However, on the third post-operative day, her symptoms did not settle further and the patient was taken back to theatre for exploration of the radial nerve due to ongoing severe pain. The radial nerve was found to be in continuity without any bruising or contusion. Distally, it was found to pass tightly against the most distal external fixator pin such that, with the elbow flexed the nerve became tightly pressed against the pin. The distal pin was removed and re-sited slightly proximal.

Post-operatively, there was complete resolution of the right arm pain and hyperalgesia. Sensation to the first webspace recovered on the second post-operative day with motor function returning more gradually. There was full recovery of radial nerve motor function at 4 months post-operatively.

## Discussion

In 1943, Seddon [2] introduced a classification of nerve injuries based on three main types of nerve fibre injury and whether there was continuity of the nerve: neurotmesis, axonotmesis and neurapraxia [2]. Neurotmesis is the most severe injury. It is a lesion in which the axon and its encapsulating connective tissue lose their continuity, resulting in Wallarian degeneration. Axonotmesis is also a degenerative lesion in which the relative continuity of an axon and its myelin sheath is lost, with preservation of its connective tissue framework. Neurapraxia is a non-degenerative lesion in which there is a local conduction block, typically of large myelinated fibres. Anatomical continuity is preserved with selective demyelination. Neurology may deteriorate as a result of progressive damage to the nerve. Neurapraxia commonly results from compression and/or ischaemia of peripheral nerves. Recovery occurs by Schwann cell repair, which occurs over weeks to months [1].

Neurapraxia can occur with or without pain. Normal sensory function is the product of an actively maintained equilibrium between neurons and their environment [3]. Any disruption of this equilibrium that results from changes in sensitivity, excitability, transmission, growth status and survival can initiate profound changes in sensory function, which explains why diverse diseases can manifest as pain.

Transient nerve compression, stretch or distortion, such as the radial neuropathy of ‘Saturday night palsy’, may result in transient nerve palsy but is typically not associated with pain. Closed nerve injuries may be managed by observation for a few months or by early exploration of the nerve. The decision is based on an assessment of the probability that the nerve has been disrupted or is under compression. Chronic nerve compression, distortion and ischaemia results in pain, termed neurostenalgia. Neurostenalgia is neuropathic pain provoked by the continuing action of the agent responsible for the lesion of the nerve, which is anatomically intact. Removal of the agent results in resolution of the pain. Chronic nerve compression can result from a wide variety of aetiologies and can equally produce a wide variety of symptoms and signs, such as muscle weakness and altered sensation in the distribution of the affected nerve. Careful history taking, examination and investigation are essential in guiding the clinician as to the cause. In all cases, the presence of intractable pain should alert the clinician to a compressive cause.

A famous case of neurostenalgia was that of the first Lord Nelson who suffered constant stump pain years after amputation of his right upper limb. Loosening and discharge of a suture round the median nerve years later resulted in a sudden resolution of the pain [1]. Birch and Strange [4] published a series of case reports describing neurostenalgia. They describe a case of compression of the lateral part of the sciatic nerve by a screw, resulting in neurostenalgia and common peroneal nerve palsy. The pain resolved 1 day post-screw removal, and within 6 weeks, the common peroneal nerve palsy had resolved. Birch [1] describes the case of an 11-year-old who suffered excruciating post-operative pain in his upper limb following vascular surgery to control major haemorrhage. A suture was found to pass through the sheath of the ulnar nerve at re-exploration. This was removed with complete resolution of the pain. He also reports the case of a 43-year-old woman who underwent ligation of the short saphenous vein. This was complicated post-operatively by foot drop and intense posture-related pain. The common peroneal nerve was found to be encircled by a suture at re-exploration. Suture removal resolved the pain. A literature review using Google Scholar and PubMed reveals isolated case reports on relief of neurostenalgia by operation years after injury [5–7].

In this case, the patient reported persistent burning pain and an exaggerated response to pin-prick sensation following application of her humeral lengthening frame 1 day post-operatively. The cause of the stimulation was confirmed as the most distal external fixator pin compressing the radial nerve because complete and immediate pain resolution occurred when the pin was re-sited. The variation in the level of symptoms was likely to have been due to a change in elbow position when the elbow was in a semi-flexed position, the nerve was compressed against the pin but with the arm fully extended the pressure was reduced. Nociceptor fibres, widely distributed in skin, muscle, joints and the walls of blood vessels, pass to fine myelinated A-delta fibres and non-myelinated C fibres. Stimulation of A-delta fibres results in sharp, well-localised pain. Stimulation of C fibres causes a dull, burning and poorly localised pain that typically is delayed [1]. This would suggest that stimulation of both C and A-delta fibres occurred in this patient.

We would recommend that clinicians consider neurostenalgia in all cases of nerve palsy associated with severe pain. This is particularly pertinent post-operatively when pain out of proportion to the procedure associated with nerve dysfunction should make the clinician consider nerve compression. A diagnosis of neurostenalgia warrants early exploration. It suggests a cause for the pain, which needs to be removed to resolve the intractable pain and prevent irreversible changes in the affected nerve.
